# Characterization of Silver Nanomaterials Derived from Marine *Streptomyces* sp. Al-Dhabi-87 and Its In Vitro Application against Multidrug Resistant and Extended-Spectrum Beta-Lactamase Clinical Pathogens

**DOI:** 10.3390/nano8050279

**Published:** 2018-04-26

**Authors:** Naif Abdullah Al-Dhabi, Abdul-Kareem Mohammed Ghilan, Mariadhas Valan Arasu

**Affiliations:** Addiriyah Chair for Environmental Studies, Department of Botany and Microbiology, College of Science, King Saud University, P.O. Box 2455, Riyadh 11451, Saudi Arabia; 436107839@student.ksu.edu.sa (A.-K.M.G.); mvalanarasu@ksu.edu.sa (M.V.A.)

**Keywords:** marine actinomycetes, nanoparticles, multidrug resistant strains, MIC

## Abstract

A novel antagonistic marine *Streptomyces* sp. Al-Dhabi-87 that was recovered from the Gulf region of Saudi Arabia was used to synthesize silver nanoparticles (NP) from the culture free extract. The produced NP were confirmed by UV-visible spectroscopy (UV-Vis), high-resolution scanning electron microscope (HRSEM), transmission electron microscope (TEM), Fourier-transform infrared spectroscopy (FTIR), Energy Dispersive Spectroscopy (EDAX), and X-ray Powder Diffraction (XRD), and broth micro dilution techniques were employed for the determination of minimum inhibitory concentrations (MIC) values. The synthesized NP was authenticated by alterations in color and wavelength scanning. HRSEM and TEM analysis confirmed that the size of the NP ranged from 10 to 17 nm and that it was spherical in shape. In addition, the FTIR spectrum revealed a variation in the band values from 500 to 3300 cm^−1^ respectively. Rietveld refinement analysis of the XRD data confirmed the size of the NP, which coincided with the results of the TEM analysis. In addition, the Riveted refinement analysis supported the TEM data. The NP documented significant activity against the wound infection microbial strains, such as *Enterococcus faecalis*, *Staphylococcus epidermidis*, and *Staphylococcus aureus*. Gram negative bacteria, such as *Pseudomonas aeruginosa*, *Klebsiella pneumonia*, and *Escherichia coli* revealed MIC values of 0.039, 0.078, and 0.152 mg/mL, respectively. The promising activity of NP towards extended-spectrum beta-lactamases *E.coli*, drug resistant *Acinetobacter baumannii*, and multidrug resistant *S. aureus* (at 0.018, 0.039, and 0.039 mg/mL, respectively) was advantageous. Overall, NP that were obtained from the novel *Streptomyces* sp. Al-Dhabi-87, with its promising antimicrobial activity towards the drug resistant pathogens, would be useful for healing infectious diseases.

## 1. Introduction

In recent years, emergence of microbial pathogens tremendously increased because of the immune compatibility of humans [1]. Therefore, to protect the humans from different diseases, many novel antibiotics and therapeutic pharmaceutical compounds with a wide level of applications were made available in the market. Reports claimed that 70% of the available antibiotics were inactive in the treatment of intracellular infections because of their reduced permeability through microbial cell walls [2]. Also, most of the commonly available functional antibiotics were amino glycosides. Beta-lactams and polyene were hydrophilic in nature, and thereby had difficulty inside the microbial cell walls. In addition, the high level of antibiotic usage triggered the development of pathogenic microbes that were resistant towards various commonly available antibiotics [3]. Among the resistant pathogens, *Staphylococcus aureus* was dominant in the world and created a serious health related disorder for humans. Specifically, some strains of *S. aureus* were resistant to methicillin, vancomycin, and multidrug antibiotics [4]. To overcome the spreading of the resistant microbial pathogens, scientists developed new antimicrobial compounds from traditional medicinal plants. They recovered novel target specific molecules from the microorganisms, especially bacteria, fungi, actinomycetes, and they developed novel drugs by combinatorial chemical biosynthesis. Despite the latest technology and the development of effective drugs, the threads from the resistant pathogens have become a serious issue [5,6,7]. However, the preparations of drug molecules with the help of nanotechnology have some advantages over the other methods of preparation. Nanoparticles (NP) play an important role in preventing the hydrophilicity barrier because of their penetration capability towards microbial cells [8,9,10,11]. Recent reports suggested that nanomaterials were prepared by using different metals such as copper (Cu), silver (Ag), titanium (Ti), gold (Au), and zinc (Zn). This is done using different methods, namely physical, chemical, and biological. Physical methods have a reduced product yield and chemical methods need a wide level of precursor molecules and solvents, and are guided to produce the toxic intermediate molecules. Biological methods are environmentally safe and have clean preparation techniques that do not produce unwanted hazardous materials during the synthesis, as well as yield a high level of chemical composition, high monodispersity, and shape/size [12,13,14].The applications of the different materials in nanotechnology antibiotic research vary with respect to the metals, and the activities also varied with respect to the infectious microorganisms [15,16]. Among the materials that were used in the synthesis of NP, Ag has been used traditionally for the preparation of biological NP for the treatment of infectious diseases. Many reports evidenced that the application of Ag inhibits the activities of microorganisms, such as bacteria, fungi viruses, insects, and nematodes. Mainly, Ag NP attack the cell wall (by enhancing the permeability of the cell wall and releasing the cell wall components), mitochondria (by affecting the ATP generation mechanism), protein (by cleaving the disulfide or sulfhydryl bonds), and DNA (by binding to the base pairs) of the pathogenic microorganisms [17]. However, actinomycete groups attracted substantial focus in research as their applications were rarely studied for the synthesis of NP [18].

Actinomycetes are Gram positive, filamentous, dry powdery in appearance, GC rich, and possess both aerial and substrate mycelium. They are capable of producing diffusible pigments thatare considered important sources among the microorganism for producing the industrially important secondary metabolites, which have various applications. They can be anti-bacterial, antifungal, antioxidant, anticancer, antidiabetic, anti-inflammatory, and antifeedant. These secondary metabolites may also be extracellular enzymes, such as cellulase, amylase, protease, lipase, xylanase, and streptokinase [19]. Reports claimed that more than 75% of commonly available antibiotics were recovered from the actinomycete groups. Among these antibiotics, the actinomycetes that were isolated from the marine environment were dominant with regards to the production of potential metabolites and active enzymes. Since the late 1980s, the identification of potential molecules from the marine environment with various biological applications has decreased because of the practical difficulties in the identification of novel molecules. Therefore, this is important, and researchers have been forced to look at the use of nanotechnological methodology for the synthesis of novel active particles from active marine environments. However, the nanomaterials synthesized using the help of marine actinomycetes, and their applications in drug resistant *S. aureus* in Saudi Arabia are rarely studied. Therefore, the present work aimed to prepare silver (Ag) nanomaterials from the identified active marine *Streptomyces* sp. Al-Dhabi-87 for the complete inhibition of multidrug resistant *S. aureus* strains.

## 2. Results

### 2.1. Antimicrobial Properties of the Marine Actinomycetes 

In the present study, marine sediment samples were collected from the Arabian Gulf regions of Saudi Arabia, namely Dammam and Al-Kohbar, for the possible detection of active antimicrobial actinomycetes. Among the identified strains, the strain Al-Dhabi-86 exhibited comparatively significant antagonistic properties against all of the tested Gram positive, Gram negative, and multiple drug resistant clinical pathogens in a cross-streak method. In addition, the spent fermentation broth also exhibited promising antimicrobial activity against the tested Gram-positive pathogens. The activity was more significant towards Gram positive bacteria than the Gram-negative pathogens. Furthermore, the biochemical, physiological, and micro-morphological characteristics revealed that the strain belonged to the actinomycete groups. Specifically, the strain was rough, white in color, and on the upper side of the plates. It was able to produce diffusible pigments when being cultivated in the Modified Nutrient Glucose (MNG) agar plates. In addition, the strain revealed various antibiotic resistant patterns towards various antibiotics. For example, it was resistant towards streptomycin. Its biochemical and physiological properties confirmed that the strain belonged to the group actinomycetes. Furthermore, 16S rRNA gene amplification and sequencing confirmed that the strain belonged to the genus *Streptomyces*. It was named *Streptomyces* sp. Al-Dhabi-87 for routine laboratory studies.

### 2.2. Synthesis and Characterization of Silver Nanoparticles

**Figure 1:**
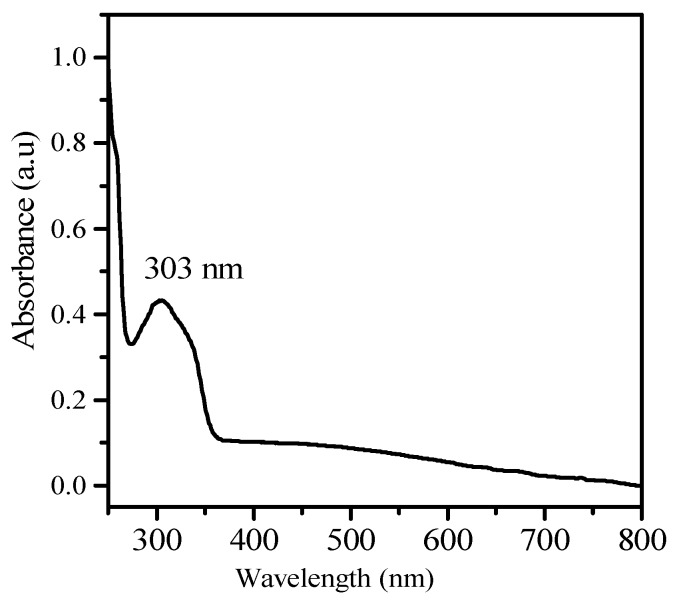
The UV spectrum of the silver nanoparticles derived from marine *Streptomyces* sp. Al-Dhabi-87.

**Figure 2:**
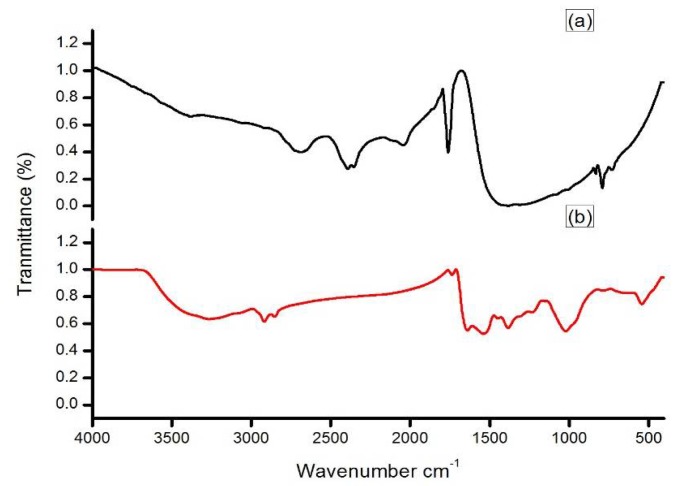
The FTIR spectrum of the silver nanoparticles derived from marine *Streptomyces* sp. Al-Dhabi-87. (**a**) FTIR spectrum of Siver nitrate (**b**) FTIR spectrum of nanoparticles.

The addition of different concentrations of sterile, ice cold aqueous solution of AgNO_3_ to the cell free, washed water samples that were triggered the synthesis of NP. The synthesis was mediated under dark conditions at 37 °C. Synthesis of the NP was confirmed by the maximum absorption spectrum obtained at a 303 nm range (Figure 1). However, among the different concentrations, 1 mM was optimal for the maximum synthesis of the NP. Furthermore, a 1 mM concentration was used for the bulk level synthesis of the NP for routine lab work. The FTIR spectrum revealed a variation in the band values of the NP, especially from 500 to 3300 cm^−1^. This indicated the presence of various functional groups. The NP bands were detected at 1050.0, 1100, 1392.5, 1500, 1800, 1920, and 2900 cm^−1^ in the FTIR spectrum (Figure 2). Overall, the presence of various functional groups clearly indicates that the synthesized NP have attached to the surface of the various extracellular components from the active actinomycete strain. 

**Figure 3:**
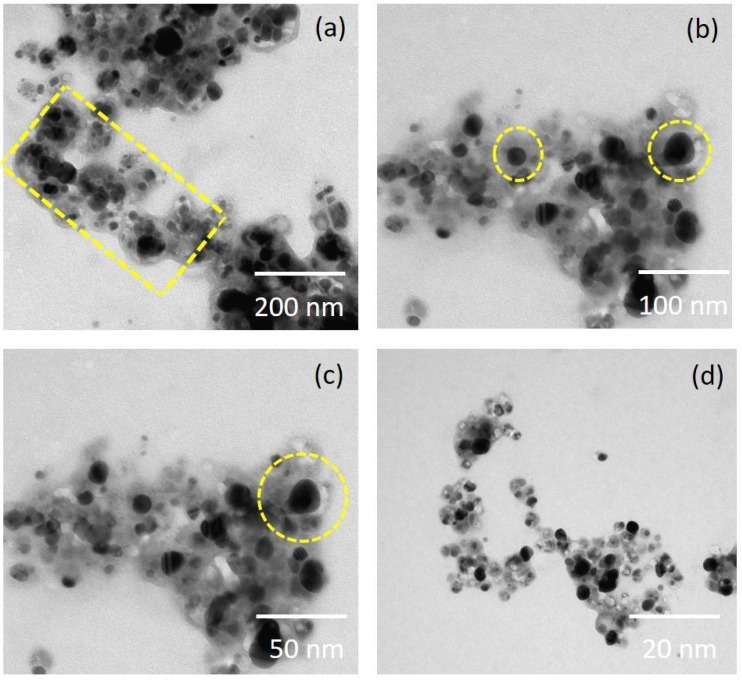
Ahigh-resolution scanning electron microscope (HRSEM) image of the silver nanoparticles derived from marine *Streptomyces* sp. Al-Dhabi-87. (**a**) HRSEM at 200 nm (**b**) HRSEM at 100 nm (**c**) HRSEM at 50 nm (**d**) HRSEM at 50 nm

**Figure 4:**
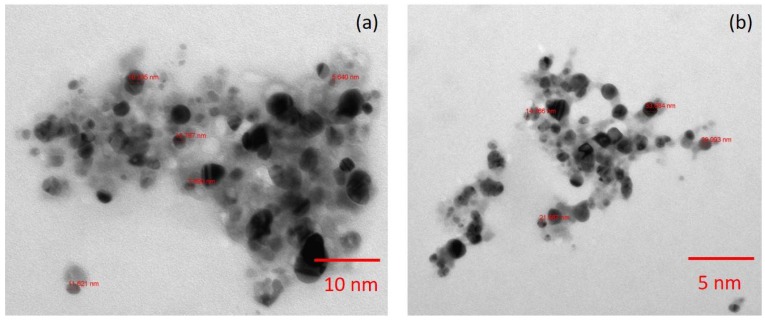
The transmission electron microscope (TEM) image of the silver nanoparticles derived from marine *Streptomyces* sp. Al-Dhabi-87. (**a**) TEM at 10 nm scale (**b**) TEM at 4 nm scale.

**Figure 5:**
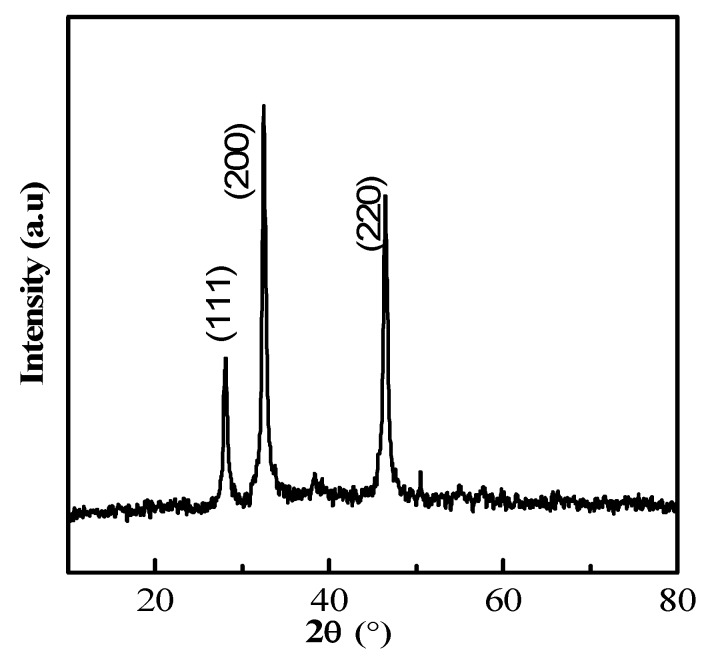
XRD profile of the silver nanoparticles derived from marine *Streptomyces* sp. Al-Dhabi-87.

**Figure 6:**
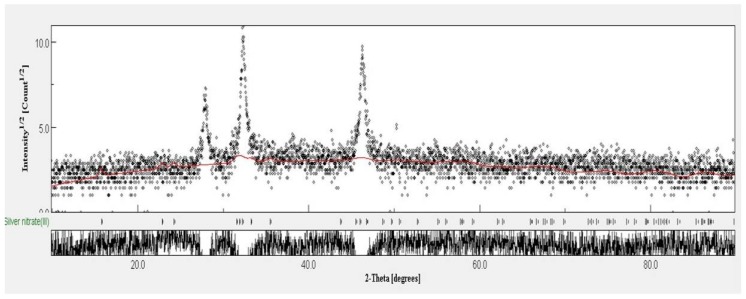
Rietveld method analysis of the XRD data of silver nanoparticles derived from marine *Streptomyces* sp. Al-Dhabi-87.

**Figure 7:**
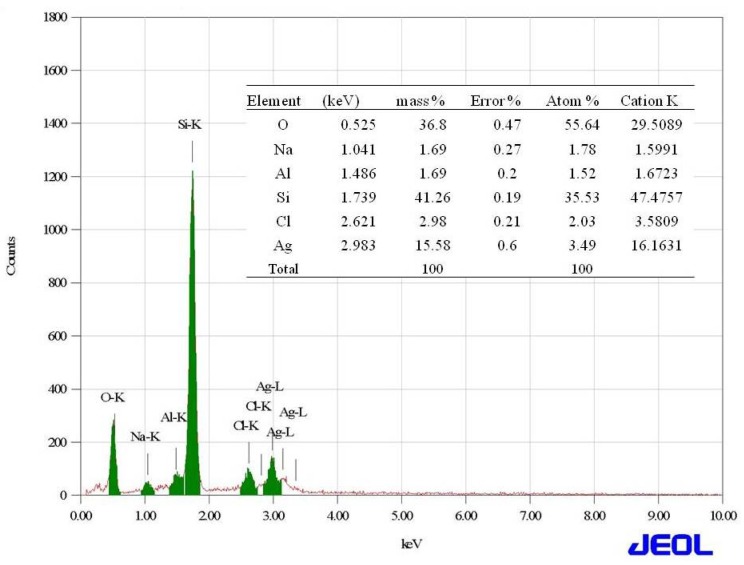
The Energy Dispersive Spectroscopy profile of the silver nanoparticles derived from marine *Streptomyces* sp. Al-Dhabi-87.

A high-resolution scanning electron microscope (HRSEM) image depicted that the synthesized NP diameter sizes were ranged from 20–50 nm (Figure 3). Also, it is interesting that the synthesized NP were spherical in shape. In the XRD studies, the selected part of the NP diffraction report clearly confirmed that the particle looks crystalline. Relatively, the transmission electron microscope (TEM) images of the synthesized NP aggregates on the copper grid, with the measuring size ranging from 10 to 17 nm (Figure 4). The XRD analysis indicated that the average particle sizes ranged from 9.7 to 17.25 nm (Figure 5). Figure 6 documented the X-ray powder diffractogram of NP from the extracellular components of the actinomycetes, including Rietveld refinement. Analysis indicated that the slight peak at 33°2Θ indicates the presence of a small amount of silver salts (ICDD PDF4 #031-1238) as a synthetic impurity. However, the size agreed with the HRSEM and TEM analysis. The stabilization of the particle size was evidenced by the image, which showed that the particles did not have contact within the aggregates. Figure 7 indicated that the NP was agglomerate in its appearance, with a slight dispersion in the other morphological surfaces.

### 2.3. In Vitro Antimicrobial Activity

#### 2.3.1. Cell Suspension Inhibition Assay

**Figure 8:**
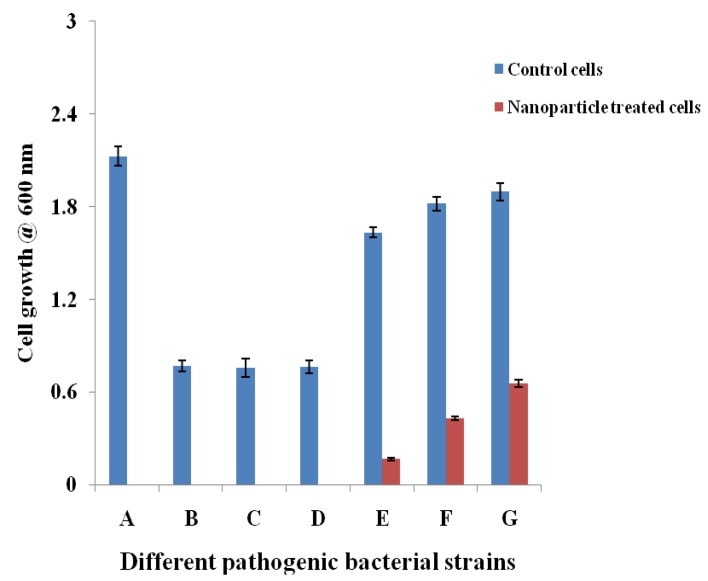
The cell suspension inhibition properties of silver nanoparticles derived from marine *Streptomyces* sp. A: *S. aureus*; B: *S. epidermidis*; C: *E. faecalis*; D: *B. subtilis*; E: *K. pneumoniae*; F: *E. coli*; and, G: *P. aeruginosa*.

The cell suspension inhibition properties of the NP were evaluated against the Gram positive and Gram-negative bacteria by measuring their growth patterns. The inhibition properties of the NP were detected by analyzing the growth pattern of the Gram positive and Gram-negative pathogens in the liquid medium using the spectrophotometer. Figure 8 revealed that the NP have a high level of inhibition properties. Specifically, the NP completely suppressed the growth of *Bacillus subtilis*, *Enterococcus faecalis*, *Staphylococcus epidermidis*, and *S. aureus* as compared with the standard strains that were cultivated without supplementation of the NP. Among the Gram-negative strains, *Klebsiella pneumoniae* was noted as the most susceptible strain to the NP treatment (90%), whereas, *Escherichia coli* and *Pseudomonas aeruginosa* documented 76% and 65% inhibition, respectively.

#### 2.3.2. Minimum Inhibitory Concentration (MIC) of the Nanoparticles

**Figure 9:**
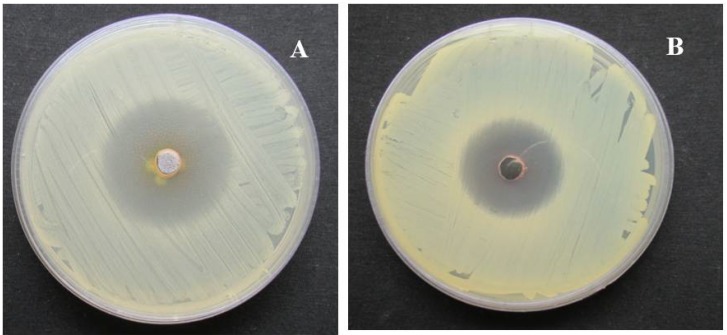
Antibacterial activity of the silver nanoparticles derived from marine *Streptomyces* sp. Al-Dhabi-87. (**A**) *Bacillus subtilis*; (**B**) *Staphylococcus aureus*.

The MIC values of the NP against standard microbial pathogens and multidrug resistant microbial pathogens are displayed in Table 1. Results indicated that the NP revealed significant activity at lower concentrations towards Gram negative bacteria. Gram positive bacteria revealed a range of MIC values from 0.039 to 1.25 mg/mL. Among the Gram-positive bacteria, *E. faecalis* and *S. aureus* documented an MIC of 0.039 mg/mL. *P. aeruginosa*, *K. pneumoniae*, and *E. coli* documented MIC values of 0.039, 0.078, and 0.152 mg/mL, respectively (Figure 9). Comparatively, NP showed better activity against the tested drug resistant pathogens, especially drug resistant *E. coli*, *Acinetobacter baumannii*, and *Proteus mirabilis.* These showed MIC values of 0.018 mg/mL. Interestingly, MRSA and drug resistant *P. aeroginosa* strains revealed an MIC value of 0.039 mg/mL. Specifically, the NP inhibited drug resistant *Enterococcus faecium* at a 0.312 mg/mL level. The MIC values of the NP were comparatively lower than the standard broad spectrum antibiotic streptomycin in the case of Gram positive bacteria.

## 3. Discussion

Nanoparticles that were synthesized from various metal ions have a large number of biological applications. The NP produced with the extracellular metabolites of the active antimicrobial compound producing strains exhibited promising activities against various Gram positive and Gram negative microbial pathogens. They inhibit the spreading of the multidrug resistant pathogenic bacteria and exhibited promising activities against the filamentous and dermatophytic fungus, as well as antiviral and anticancer properties. Therefore, the present study aimed to produce Ag NP from the extracellular extracts of the promising marine *Streptomyces* sp. Al-Dhabi-87 that was recovered from the Arabian Gulf regions of Saudi Arabia. Results indicate a significant amount of the NP that were synthesized at lower concentrations (1 mM) of the Ag. However, Prakash and Thiagarajan (2012) claimed that maximum yield of the NP was obtained at 1.5 mM level of Ag metals [20]. The synthesis of the NP was stimulated with the help of the active substrates accumulated on the cell wall of the actinomycetes. These directly induce the release of the extracellular enzymes, which enhance the reduction of metal ions to NP. In the present study, the initial formations of the Ag NP were confirmed by the change of color from colorless to brown, because of the possible reduction of the Ag ions. Similarly, Sastry et al. (2003) observed the change in color during the extracellular synthesis of the NP from the actinomycete strains [21]. Also, the recent study conducted by Deepa et al., (2013) proved that the fast synthesis of the Ag NP by the water extract of the actinomycetes were because of the presence of a diverse level of metabolites and enzymes on the surface of the cell wall [22]. Interestingly, until now, no evidenced report described the complete mechanism of the synthesis of the NP from the microbial route because the microbes have unpredictable mechanisms in interacting with metal ions. The synthesis of the NP could be either at the intracellular level or the extracellular level [23,24,25,26]. The present study proved that the synthesis of the NP was extracellular in nature. The present study was similar to the report of Karthik et al. (2014) [27]. Korbekandi et al. (2009) claimed that the reduction of silver nitrate by *Streptomyces* sp. LK-3 could be due to the enhancing action of the extracellular nitrate reductase enzyme [28]. As reported by other studies, the synthesized NP were characterized by using techniques, such as UV-visible spectroscopy (UV-Vis), XRD, HRSEM, TEM, and FTIR, which confirmed that the particle size was in the nano range [29,30,31,32,33]. The spectral analysis also confirmed the NP synthesis was the advantage of this study. The synthesis of the NP by a biological route was evidenced with the help of HRSEM and TEM images [33]. Also, the standard size of the NP ranged from 5 to 100 nm, with different shapes, such as square, spherical, round, and polydisperse. Similarly, the HRSEM and TEM images confirmed thatthe sizes ranged from 10 to 17 nm.

Recently, the use of nanomaterials that were synthesized from the biological route to stop the spread of the drug resistant strains, which emerged from hospitals, has attracted the interest of researchers [34]. Specifically, the participation of the marine actinomycetes in the field of nonmaterial synthesis gained the interest of drug companies because of their specific activities to target drug resistant Gram positive and Gram negative pathogens. To confirm this statement, this study also showed that the synthesized NP significantly inhibited the growth of the Gram positive pathogens that caused wound infections and drug resistant pathogens. Among the tested drug resistant strains, *A. baumannii* revealed the lowest MIC values (0.018 mg/mL), followed by multidrug resistant *S. aureus* (0.039 mg/mL). Recently, Iniyan et al. (2016) claimed that the silver chloride NP that were obtained from the *Streptomyces exfoliatus* ICN25 of the mangrove *Rhizophora mucronata* showed positive inhibition activity against the methicillin-resistant *S. aureus*, *P. aeruginosa*, *K. pneumonia*,and *E. coli* at 1 µg/mL level [35]. It is predicted that the antimicrobial activity of the NP might be due to the action of the particles to the cells, which triggers the leakage of the intracellular components and alterations in the cellular structure [36]. Many reports claimed that the Gram positive microbial strains are commonly resistant to the NP, especially the peptidoglycan layer of the Gram positive bacteria (*S. aureus*). This layer had a thickness of approximately 80 nm and linked with the group of heteropolymeric polysaccharides, such as teichoic and teichuronic acids [37,38].

## 4. Materials and Methods

### 4.1. Chemicals and Reagents

Silver nitrate was purchased from Himedia, Mumbai, India. Microbial cultivation media, glucose, and other solvents were purchased from Sigma Aldrich (St. Louis, MO, USA). Streptomycin was procured from Himedia, Mumbai, India.

### 4.2. Isolation and Characterization of Antagonistic Actinomycetes

Marine sediment soil samples collected from the bank of the Arabian Ocean at the Dammam of Saudi Arabia were used for the isolation of a novel actinomycete strain. Briefly, the samples were serially diluted with sterile distilled water, and were spread on the starch casein agar medium supplemented with actidione, nalidixic acid, and streptomycin. They were kept at 30 °C for two weeks. These antibiotics were used for the selective isolation of the actinomycetes. After the incubation, the suspected actinomycetes were selected and purified using ISP2 medium and stored in the refrigerator for the routine lab studies. For antimicrobial screening, modified nutrient glucose agar medium was prepared, and the incubatory properties of the strains were checked by the cross-streak method, i.e., by growing both Gram negative and Gram positive bacterial strains that were perpendicular to the actinomycete strains. After incubation at 37 °C for 17 h, the active strains were labeled and identified for the NP synthesis. Among the 51 screened actinomycete strains, strain 34 exhibited promising activities against the pathogens and was hence selected for the identification studies. The biochemical, physiological, and antimicrobial sensitivity pattern of the strain was studied by following the standard methodology. Furthermore, the selected strain was confirmed by 16S rRNA amplification and sequencing.

### 4.3. Synthesis and Characterization of Silver Nanoparticles

For the preparation of Ag NP, the actinomycetes strain was cultivated in an MNG broth for 14 days at 30°C and 150 rpm. After cultivating the actinomycetes in the fermentation liquid medium containing the nutritional components, the actinomycetes cells were centrifuged at 15,000 rpm for 15 min at 4°C. The separated cell pellets were collected and washed thoroughly with sterile distilled water to remove the fermented MNG broth. Next, the broth free cells were mixed with sterile distilled water and were kept at shaking conditions for 1 h. Next, the cells were removed from the suspension, and different concentrations of silver nitrate (1, 2, 3, 4, and 5 mM) were mixed with the supernatant and kept at 37 °C for 48 h. Control experiments were conducted with plain distilled water. The change in the color of the supernatant indicated the synthesis of the NP. The synthesized NP was centrifuged at 15,000 rpm for 30 min, and the pellets were collected and analyzed for the confirmation of NP. The maximum absorption spectrum of the NP was checked by scanning with a UV-Vis spectrophotometer (Double Beam Shimadzu UV-2600, Tokyo, Japan) from 200 to 700 nm. The crystalline nature of the NP was determined by XRD study using the MiniFlex-600 (Rigaku, Tokyo, Japan) together with the Cu Kα.The operating condition of the machine was 40 kV power and 30 mA current. The ratio of Cu/kα radiation (λ = 1.5418 Å) was in the range of 20°–80° in 2θ angles. The Debye–Scherrer mathematical formula was used for the calculation of the average particle size (*D* = kλ/β_1/2_cos θ.38) [39,40]. The functional components of the synthesized NP were determined by analyzing the FTIR spectrum. Briefly, 10 mg of the dry powdered samples were mixed and were then coated with KBr pellets for measuring a wavelength range of 4000 and 400 cm^−1^ at a resolution of 4 cm^−1^ with a Bruker TENSOR 27 Spectrometer (Bruker, Tokya, Japan). 

Furthermore, the functional groups were compared with the reported standard spectrum. The Rietveld refinement study has been performed with the powder of the standard silver nitrate from the National Institute of Standards and Technology (NIST) as the authenticated reference compound. It is noted that the instrumental peak broadening was different for the two diffractometers. The HRSEM image was taken to determine the size, shape, and morphology of the produced NP.Briefly, the powdered samples in the suspension were carbon coated on the copper coated grid, and the excess suspension was removed and then dried using a shard dryer. After that, the image was taken using the FEHRSEM, JEOLJSM-7600F (Jeol, Tokyo, Japan) at the voltage of 20,000 V. The X-ray detector (JED-2200 series) (Jeol, Tokyo, Japan) was fitted with 20,000 V for determining the elemental analysis energy-dispersive X-ray analysis. In addition, the exact size and the outer morphology of the synthesized NP were noted using a high-resolution transmission electron microscope (HRTEM, JEOL JSM-2100F) (Jeol, Tokyo, Japan), operating at 20,000 and 5 kV. The spectral analyses were performed at the central laboratory of the College of Science in King Saud University.

### 4.4. In Vitro Antimicrobial Activity

#### 4.4.1. Microbial Pathogens

Gram positive bacteria, such as *B. subtilis*, *E. faecalis* (ATCC 29212), *S. epidermidis* (ATCC 12228), and *S. aureus* (ATCC 29213), and Gram-negative bacteria, such as *P. aeruginosa* (ATCC27853), *K. pneumoniae* (ATCC70063), and *E. coli* (ATCC25922) were obtained from the American Type Culture Collection. Drug resistant clinical strains, such as *E. coli* (ESBL 4345), *A. baumannii* (MDR 4414), *P. mirabilis* (DR 4753), *E. faecium* (VRETC 773), *E. coli* (ATCC 35218), and multidrug resistant *S. aureus* (WC 25 V 880854) were kindly gifted from the Department of Clinical Microbiology at the King Khalid University Hospital and the National Guard Hospital, Riyadh, Saudi Arabia. 

#### 4.4.2. Cell Suspension Inhibition Assay

The cell suspension inhibition assay was evaluated by mixing the NP suspension with the active growing cells. Briefly, 100 microliters of mid log phase suspension of different Gram positive, Gram negative pathogens, and multi drug resistant clinical pathogens were transferred into 5 mL of sterile Muller–Hinton (MH) broth and 500 microliters of the synthesized NP. Next, the mixture was incubated at 37 °C for 17 h and 100 rpm in a shaker incubator. Separately, bacterial cells were incubated alone and were considered as the positive control. After the incubation, the cells were centrifuged to separate the broth and re-suspend the cells with sterile distilled water. The killing effect of the NP was determined by measuring the growth of the cells using a spectrophotometer at 600 nm. 

#### 4.4.3. Determination of the MIC of the Nanoparticles

The liquid broth dilution method with a double fold dilution technique was followed for the determination of the MIC of the synthesized NP. In detail, 20 mg of the NP was sonicated with 100µL of sterile distilled water, and the suspension was used for the evaluation of the antimicrobial activities. The experiment was performed in the 200 microliter holding capacity of sterile 96-well microtiter plates. Briefly, the 200 microliter mixture contained sterile MH broth (185 µL), a measured concentration of NP (10 µL), and different bacterial pathogenic strains (5 µL). The bacterial pathogenic strains were cultivated fresh and the final concentrations of the pathogens were approximately 10^7^ CFU/mL. The plate was incubated at 37 °C for 17 h. After incubation, 5 µL of the cell suspension was spotted on the MH agar plates and was incubated at 37 °C for 17 h for the visible growth of the bacterial strains [41]. Separately, standard antibiotic streptomycin was used as a positive control [42]. The spot without cell growth was identified as the concentration in which the bacteria were completely killed. The experiment was conducted three times for the confirmation of the MIC of the NP [43].

## 5. Conclusions

In summary, the present study highlighted the identification and the characterization of novel marine actinomycetes that are capable of synthesizing novel Ag metal NP. The biochemical, physiological, and molecular level properties confirmed that the strain belonged to the *Streptomyce* sp. The cell free washed extracts quickly act as the mediator for the synthesis of Ag NP. The synthesized NP were confirmed by various analytical and spectroscopic techniques, such as UV, FTIR, HRSEM, TEM, EDX, and XRD respectively. The hydrophilic and hydrophobic small metabolites attached on the surface of the actinomycetes could act as the mediator for the enhanced reduction of Ag ions, and thereby stimulated the synthesis of the NP. XRD analysis revealed average particle sizes from 9.7 to 17.25 nm. Alternatively, the TEM images of the synthesized NP aggregates on the copper grid measured sizes ranging from 10 to 17 nm. The FTIR spectrum revealed variation in the band values from 500 to 3300 cm^−1^, respectively. Rietveld refinement analysis of the XRD data confirmed the size of the NP, which coincided with the results of the TEM analysis. Interestingly, the produced NP documented promising antimicrobial activity against the wound infection pathogens, such as *B. subtilis*, *E. faecalis*, *S. epidermidis*, and multidrug resistant *S. aureus*. The antimicrobial killing effect of the NP at lower concentrations is similar to commercial antibiotics in its advantage in the application of pharmaceutical industries. In addition, the NP showed promising activity towards the extended-spectrum beta-lactamase (ESBL) *E. coli* strains, and this was an added advantage. Therefore, the promising marine strain could be ideal for the development of nano-medicine for the treatment of infectious pathogens. 

## Figures and Tables

**Table 1:** In vitro antimicrobial activities of silver nanoparticles derived from marine *Streptomyces* sp. Al-Dhabi-87.

Pathogens	MIC Values (mg/mL)
**Gram positive**	
*Bacillus subtilis*	1.25
*Enterococcus faecalis* (ATCC 29212)	0.039
*Staphylococcus epidermidis* (ATCC 12228)	1.25
*Staphylococcus aureus* (ATCC 29213)	0.039
**Gram negative**	
*Pseudomonas aeruginosa* (ATCC 27853)	0.039
*Klebsiella pneumoniae* (ATCC 0063)	0.078
*Escherichia coli* (ATCC 25922)	0.152
**Drug resistant strains**	
*Escherichia coli* (ESBL 4345)	0.018
*Acinetobacter baumannii* (MDR 4414)	0.039
*Pseudomonsa aeroginosa* (MDR 4406)	0.039
*Acinetobacter baumannii* (MDR 4474)	1.25
*Proteus mirabilis* (DR 4753)	0.018
*Acinetobacter baumannii* (4414)	0.312
*Acinetobacter baumannii* (MDR 4273)	0.156
*Acinetobacter baumannii* (MDR 7077)	0.312
*Acinetobacter baumannii* (MRO 3964)	0.018
*Enterococcus faecium* (VRETC 773)	0.312
*Enterococcus faecium* (VRE UR 83198))	0.312
*Multidrug Resistant Staphylococcus aureus* (WC 25 V 880854)	0.039
*Multidrug Resistant Staphylococcus aureus* (V 552)	0.039
*Staphylococcus aureus* (ATCC 43300)	0.039
*Staphylococcus aureus* (TC 7692)	0.039
*Escherichia coli* (ATCC 35218)	0.076

MIC: minimum inhibitory concentration; ESBL: extended-spectrum beta-lactamases; MDR: multidrug resistant; ATCC: American type culture collection.
